# Visit-to-visit variability in triglyceride-glucose index and diabetes: A 9-year prospective study in the Kailuan Study

**DOI:** 10.3389/fendo.2022.1054741

**Published:** 2022-12-02

**Authors:** Xianxuan Wang, Yanjuan Chen, Zegui Huang, Zefeng Cai, Xinran Yu, Zekai Chen, Linyao Li, Guanzhi Chen, Kuangyi Wu, Huancong Zheng, Shouling Wu, Youren Chen

**Affiliations:** ^1^ Department of Cardiology, Second Affiliated Hospital of Shantou University Medical College, Shantou, Guangdong, China; ^2^ Department of Endocrinology, Second Affiliated Hospital of Shantou University Medical College, Shantou, Guangdong, China; ^3^ Department of Anesthesiology, North China University of Science and Technology, Tangshan, China; ^4^ Department of Epidemiology, University Medical Center Groningen, University of Groningen, Groningen, Netherlands; ^5^ Department of Plastic Surgery, Chongqing Huamei Plastic Surgery Hospital, Chongqing, China; ^6^ Second Clinical College, China Medical University, Shenyang, China; ^7^ Department of Cardiology, Kailuan General Hospital, Tangshan, China

**Keywords:** triglyceride-glucose index, variability, diabetes mellitus, cohort study, insulin resistance

## Abstract

**Instruction/Aims:**

It is unknown whether variability in the triglyceride-glucose index (TyG-index) is associated with the risk of diabetes. Here, we sought to characterize the relationship between TyG-index variability and incident diabetes.

**Methods:**

We performed a prospective study of 48,013 participants in the Kailuan Study who did not have diabetes. The TyG-index was calculated as ln [triglyceride (TG, mg/dL) concentration × fasting blood glucose concentration (FBG, mg/dL)/2]. The TyG-index variability was assessed using the standard deviation (SD) of three TyG-index values that were calculated during 2006/07, 2008/09, and 2010/11. We used the Cox proportional hazard models to analyze the effect of TyG-index variability on incident diabetes.

**Results:**

A total of 4,055 participants were newly diagnosed with diabetes during the study period of 8.95 years (95% confidence interval (CI) 8.48–9.29 years). After adjustment for confounding factors, participants in the highest and second-highest quartiles had significantly higher risks of new-onset diabetes *versus* the lowest quartile, with hazard ratios (95% CIs) of 1.18 (1.08–1.29) and 1.13 (1.03–1.24), respectively (*P* trend< 0.05). These higher risks remained after further adjustment for the baseline TyG-index.

**Conclusions:**

A substantial fluctuation in TyG-index is associated with a higher risk of diabetes in the Chinese population, implying that it is important to maintain a normal and consistent TyG-index.

## Introduction

Owing to socioeconomic advances and rising standards of living, the prevalence of diabetes mellitus in China has risen sharply over the past four decades, from 0.67% in 1980 to 12.8% in 2018 (1, 2). There were 140.9 million people in China with diabetes in 2019, and in 2045, the number is predicted to reach 174.4 million (3). Furthermore, diabetes is a risk factor for cardiocerebrovascular events, renal dysfunction, and overall morality (4–7), which have a major impact on society and the economy. Insulin resistance is a key pathogenetic feature of diabetes (8, 9), which is characterized by various metabolic disorders, including hyperglycemia and hypertriglyceridemia (10). Thus, it is essential to identify and control insulin resistance early to prevent diabetes.

The assessment of insulin resistance in the clinical setting is challenging because the gold standard method of the euglycemic clamp is expensive and relatively complex (11). Instead, the triglyceride-glucose (TyG) index, which is the product of the fasting blood glucose (FBG) and the fasting triglyceride (TG) concentration, has become established as reliable surrogate marker of insulin resistance (12, 13). Several studies have shown a link between a high TyG-index and diabetes (14–16). Furthermore, cohort studies conducted in European, Korean, and Chinese populations have revealed that a high TyG-index level is also associated with subsequent incident cardiovascular disease (CVD) (17–19). Although most previous studies of this index considered single measurements, it can be affected by several factors, such as age, diet, and exercise (20). The variability of the TyG index can reflect the long-term level of fluctuation (21). Therefore, in the present study, we aimed to test the hypothesis that high TyG-index variability is associated with the risk of diabetes-related outcomes in the Chinese population.

## Materials and methods

### Study sample

**Figure 1:**
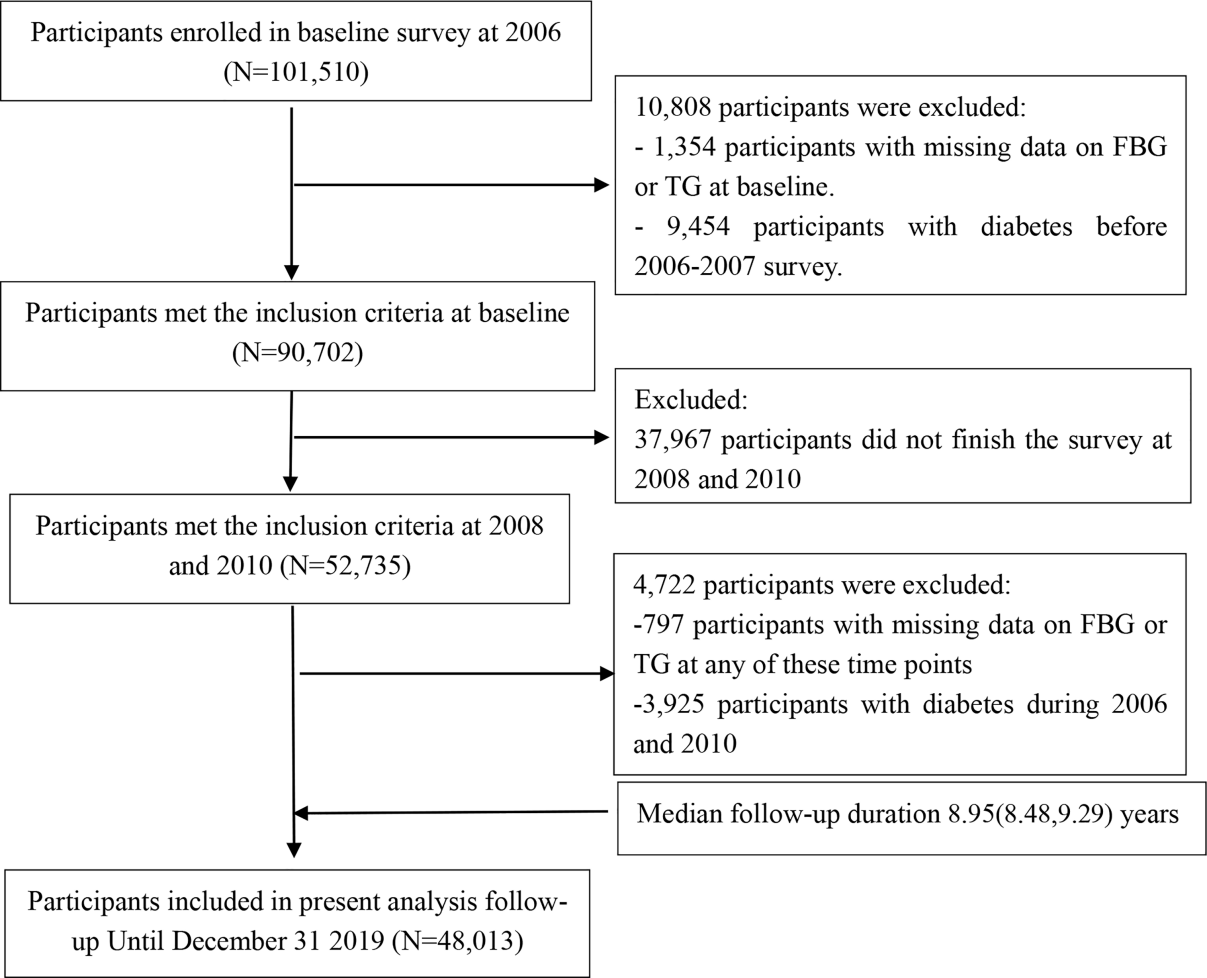
Flow chart for the inclusion of participants in the study.

We studied data from the Kailuan Study, an ongoing prospective cohort study (22). This comprised information regarding 101,150 individuals who were enrolled to participate in a biennial questionnaire-based interview, which covered their demographic characteristics, medical history, and lifestyle; to undergo clinical examinations; and to undergo the measurement of laboratory parameters between 2006 and 2007. For the present study, the participants were required to have undergone two consecutive medical examinations during 2008/09 and 2010/11 to be eligible. Participants were excluded if they had diabetes in or prior to 2010, or if their FBG or TG data were missing for any of the examinations. After the application of these criteria, 48,013 participants remained for enrollment in the present study (**Figure 1**). The first survey, during 2006/07, was defined as the baseline survey, and the third survey (2010/11) as the starting point of the follow-up period.

All the participants gave their written informed consent and the study protocol was approved by the Ethics Committee of the Kailuan General Hospital (approval number: 2006-05).

### TyG index and the calculation of TyG index variability

The TyG index was calculated as ln [TG (mg/dL) × FBG (mg/dL)/2] (23). TyG index variability was defined as the intra-individual variability of the TyG index, calculated using data collected during the three physical examinations. Four indices of variability were used:

(1) standard deviation (SD): SD = $\sqrt{\frac{1}{n-1}\sum_{\text{i}=1}^{\text{n}}{\left(x_{i}-\bar{x}\right)}^{2}}$ ;

(2) coefficient of variation (CV): CV = (SD/mean × 100%);

(3) variation independent of the mean (VIM) (24, 25): VIM = SD/mean^χ^, where “mean” is the average of the mean TyG index values for the participants, and χ is derived from non-linear regression analysis in the PROC NLIN procedure of the SAS package (SAS Institute Inc., Cary, NC, USA);

(4) average real variability (ARV) (21): $ARV=\frac{1}{N-1}\sum_{K=1}^{N-1}\left|Value_{K+1}-Value_{k}\right|$ .; and

(5) Slope of the TyG index change: regression lines were created using the three sets of TyG index data, and the slope of this regression line represented the overall trend in TyG index. This was used as an index of the long-term change in the TyG index. In the present study, a slope of the change in TyG index > 0 indicated overall positive variation, and a slope ≤0 indicated overall negative variation.

As previously described (26, 27), we placed the participants into four groups according to quartiles of the baseline SD of the TyG index: a Q1 group,<0.18; a Q2 group, 0.18–0.30; a Q3 group, 0.30–0.44; and a Q4 group, ≥0.44.

### Outcome events

The outcome of the present study was new-onset diabetes, which has been defined previously in detail (28). Briefly, diabetes (29) was defined using an FBG of ≥7.0 mmol/L, the use of glucose-lowering drugs, or a self-reported history of diabetes. Participants were followed from their third examination, during 2010/11, to the first of the date on which diabetes was first diagnosed, the date of death or December 31, 2019.

### Assessment of covariates

The demographic data (e.g., age, sex, and educational background), lifestyle (smoking, alcohol consumption, and physical activity habits) and medical history (hypertension and diabetes) of the participants were collected using questionnaires completed at face-to-face interviews. BMI was calculated as body mass (kg) divided by the square of height (m). Height, body mass, and blood pressure were measured by trained physicians using a standardized protocol.

Participants were instructed to visit the testing site in the morning after at least 8 hours of fasting and blood samples were collected from a cephalic vein by a trained laboratory technician. An automatic biochemical analyzer (7600-020, Hitachi, Tokyo, Japan) was used to measure the FBG, TG, low-density lipoprotein-cholesterol (LDL-C), high-density lipoprotein-cholesterol (HDL-C), and high-sensitivity C-reactive protein (hs-CRP) concentrations. A current smoker was defined as someone who had smoked a mean of ≥ 1 cigarette per day during the preceding year, and participants were categorized as non-smokers or current smokers. An alcohol consumer was defined as someone who drank a mean of ≥ 100 mL of alcohol per day for at least the preceding year, and participants were categorized as non-drinkers or current drinkers. Participants were categorized as undertaking physical exercise if they performed exercise ≥ 3 times per week for ≥ 30 min on each occasion (30). Education was classified as high school or above *vs*. below high school level. Hypertension (31) was defined as a blood pressure ≥140/90 mmHg, the use of antihypertensive medication, or a self-reported history of hypertension.

### Statistical analysis

Normally distributed, continuous data are expressed as mean ± standard deviation (x̅ ± s) and non-normally distributed data as median (25%, 75% percentile), and were analyzed using one-way ANOVA or the Kruskal-Wallis rank sum test, respectively. Categorical data are expressed as absolute number and percentage and were analyzed using the chi-square test.

We used the Kaplan–Meier method to calculate the cumulative incidence of the primary outcome in each group and then compared the groups using the log-rank test. We also used univariate and multivariate Cox regression models to identify potential risk factors for diabetes. The relationship between TyG index variability and diabetes was characterized using Cox proportional hazards regression models. In model 1, we adjusted for age (continuous) and sex (categorical) at baseline. In model 2, we further adjusted for LDL-C (continuous), HDL-C (continuous), hs-CRP (continuous), BMI (continuous), smoking status (categorical, yes/no), alcohol consumption status (categorical, yes/no), physical exercise habits (categorical, yes/no), educational level (categorical), hypertension (categorical, yes/no), and the use of lipid-lowering medication (categorical, yes/no) at the start of the follow-up period. In model 3, we further adjusted for the TyG index at baseline.

We further conducted stratified analyses by the sex, age, and slope of the change in the TyG index of the participants. Several sensitivity analyses were conducted as follows: (1) after the exclusion of participants in whom diabetes developed within the first year of follow-up; (2) after the exclusion of participants who were taking lipid-lowering or antihypertensive medication; (3) after the exclusion of participants with a TG concentration ≥ 2.3 mmol/L at baseline; (4) adjusting for the baseline TG and FBG concentrations and without the inclusion of the baseline TyG-index; and (5) using other indices of TyG-index variability (ARV, CV, and VIM) instead of SD. We also repeated the analyses using Cox proportional hazards models. A two-sided *P<* 0.05 was considered to be statistically significant. We used SAS (version 9.4, SAS Institute Inc.) for the statistical analyses.

## Results

### Baseline characteristics of the study sample

A total of 48,013 participants were selected for the study. Their mean age was 48.78 ± 12.05 years and 36,356 (75.72%) were male. Compared with the Q1 group, the Q2 and Q3 groups had much higher BMI, SBP, DBP, TG, FBG, hs-CRP; and had higher prevalences of smoking, drinking, and hypertension (*P*<0.01; **Table 1**).

**Table 1:** Baseline characteristics of participants by TyG index variability quartiles (SD).

	Total	Q1	Q2	Q3	Q4	*P*
Participants	48013	12003	12003	12004	12003	
Age(years)	48.78 ± 12.05	50.09 ± 12.31	49.36 ± 12.09	48.85 ± 12.05	46.82 ± 11.51	<.01
Male, N (%)	36356 (75.72)	8805(73.36)	8978(74.80)	8989 (74.88)	9584 (79.85)	<.01
BMI (kg/m^2^)	24.84 ± 3.12	24.76 ± 3.15	24.81 ± 3.18	24.87 ± 3.15	24.92 ± 3.00	<.01
SBP (mmHg)	128.55 ± 16.75	128.36 ± 16.93	128.44 ± 16.91	128.66 ± 16.92	128.73 ± 16.22	0.07
DBP (mmHg)	83.19 ± 9.18	82.82 ± 9.15	82.94 ± 9.15	83.25 ± 9.25	83.77 ± 9.15	<.01
HDL-C (mmol/L)	1.54 ± 0.32	1.55 ± 0.32	1.55 ± 0.32	1.54 ± 0.31	1.53 ± 0.32	<.01
LDL-C (mmol/L)	2.48 ± 0.63	2.50 ± 0.63	2.50 ± 0.63	2.48 ± 0.64	2.44 ± 0.63	<.01
FBG (mmol/L)	5.24 ± 0.52	5.09 ± 0.32	5.14 ± 0.60	5.35 ± 0.38	5.49 ± 0.47	<.01
TG (mmol/L)	1.30 (0.96-1.87)	1.21(0.88-1.58)	1.27(0.95-1.81)	1.32 (1.01–1.75)	1.69 (1.14-2.48)	<0.01
Hs-CRP (mg/L)	1.42 (0.76-2.83)	1.37 (0.73-2.70)	1.40 (0.76-2.71)	1.43 (0.77-2.92)	1.46 (0.78-3.06)	<0.01
TyG index_2006_	8.55 ± 0.63	8.49 ± 0.50	8.52 ± 0.54	8.52 ± 0.60	8.71 ± 0.77	<.01
TyG index_2008_	8.57 ± 0.62	8.51 ± 0.50	8.49 ± 0.53	8.56 ± 0.60	8.69 ± 0.80	<.01
TyG index_2010_	8.61 ± 0.61	8.50 ± 0.51	8.56 ± 0.53	8.64 ± 0.59	8.90 ± 0.75	<.01
Smoking, N (%)	18360 (38.24)	4296.0 (35.79)	4423 (36.85)	4680 (38.99)	4961 (41.33)	<.01
Drinking, N (%)	16957 (35.32)	3948 (32.89)	4101 (34.17)	4188 (34.89)	4720 (39.32)	<.01
Physical activity, N (%)	6906 (14.38)	1944 (16.20)	1817 (15.14)	1697 (14.14)	1448 (12.06)	<.01
Hypertension, N (%)	22072 (45.97)	5391 (44.91)	5444 (45.36)	5563 (46.34)	5674 (47.27)	<.01
Antihypertensive drugs, N (%)	6944 (14.46)	1654 (13.78)	1715 (14.29)	1738 (14.48)	1837 (15.30)	<.01
Lipid-lowering drugs, N (%)	728 (1.52)	186 (1.55)	178 (1.48)	165 (1.37)	199 (1.66)	0.33
High school or above, N (%)	6217 (12.95)	1599 (13.32)	1606 (13.38)	1583 (13.19)	1429 (11.91)	<.01

P, comparison of baseline characteristics between different TyG index variability groups.

TyG index, triglyceride-glucose index; BMI, body mass index; SBP, systolic blood pressure; DBP, diastolic blood pressure; TG, triglyceride; HDL-C, high-density lipoprotein cholesterol; LDL-C, low-density lipoprotein cholesterol; FBG, fasting blood glucose; hs-CRP, high-sensitivity C reactive protein.

### Results of the univariate and multivariate Cox regression analyses to identify risk factors for diabetes

Univariate Cox proportional-hazards regression showed that TyG index variability, age, sex, SBP, DBP, TyG-index, LDL-C, HDL-C, hs-CRP, BMI, smoking status, educational level, physical activity habits, hypertension, and the use of lipid-lowering drugs were significantly associated with diabetes (*P*< 0.05, **Table 2**).

**Table 2:** Risk factors for diabetes were analyzed by univariate and multivariate Cox regression analysis.

	Univariate Cox regression analyses		Multivariate Cox regression analyses	
	HR (95%CI)	*P* value	HR (95%CI)	*P* value
TyG index variability	1.08 (1.07,1.10)	<0.01	1.06 (1.03,1.09)	<0.01
Age	1.01 (1.01,1.02)	<0.01	1.01 (1.00,1.01)	<0.01
Gender	1.22 (1.13,1.32)	<0.01	0.98 (0.90,1.07)	0.68
BMI	1.16 (1.15,1.17)	<0.01	1.12 (1.10,1.13)	<0.01
SBP	1.04 (1.04,1.05)	<0.01	/	/
DBP	1.06 (1.05,1.06)	<0.01	/	/
HDL-C	0.63 (0.56,0.70)	<0.01	0.82 (0.74,0.92)	<0.01
LDL-C	1.24 (1.19,1.30)	<0.01	1.11 (1.06,1.16)	<0.01
hs-CRP	1.03 (1.02,1.03)	<0.01	1.01 (1.00,1.02)	<0.01
TyG index_2006_	1.95 (1.87,2.05)	<0.01	1.82 (1.72,1.91)	<0.01
Current smoking	1.06 (1.00,1.13)	<0.01	1.03 (0.95,1.11)	0.50
Current drinker	1.02 (0.95,1.08)	0.12	0.96 (0.89,1.04)	0.31
Physical activity	0.94 (0.90,0.98)	<0.01	0.94 (0.86,1.03)	0.17
Hypertension	1.90 (1.79,2.02)	<0.01	1.29 (1.21,1.38)	<0.01
education	0.74 (0.69,0.78)	<0.01	0.80 (0.75,0.85)	<0.01
Lipid-lowering drugs	1.85 (1.53,2.24)	<0.01	1.22 (1.00,1.48)	0.04

### Relationship between TyG-index variability and incident diabetes

**Figure 2:**
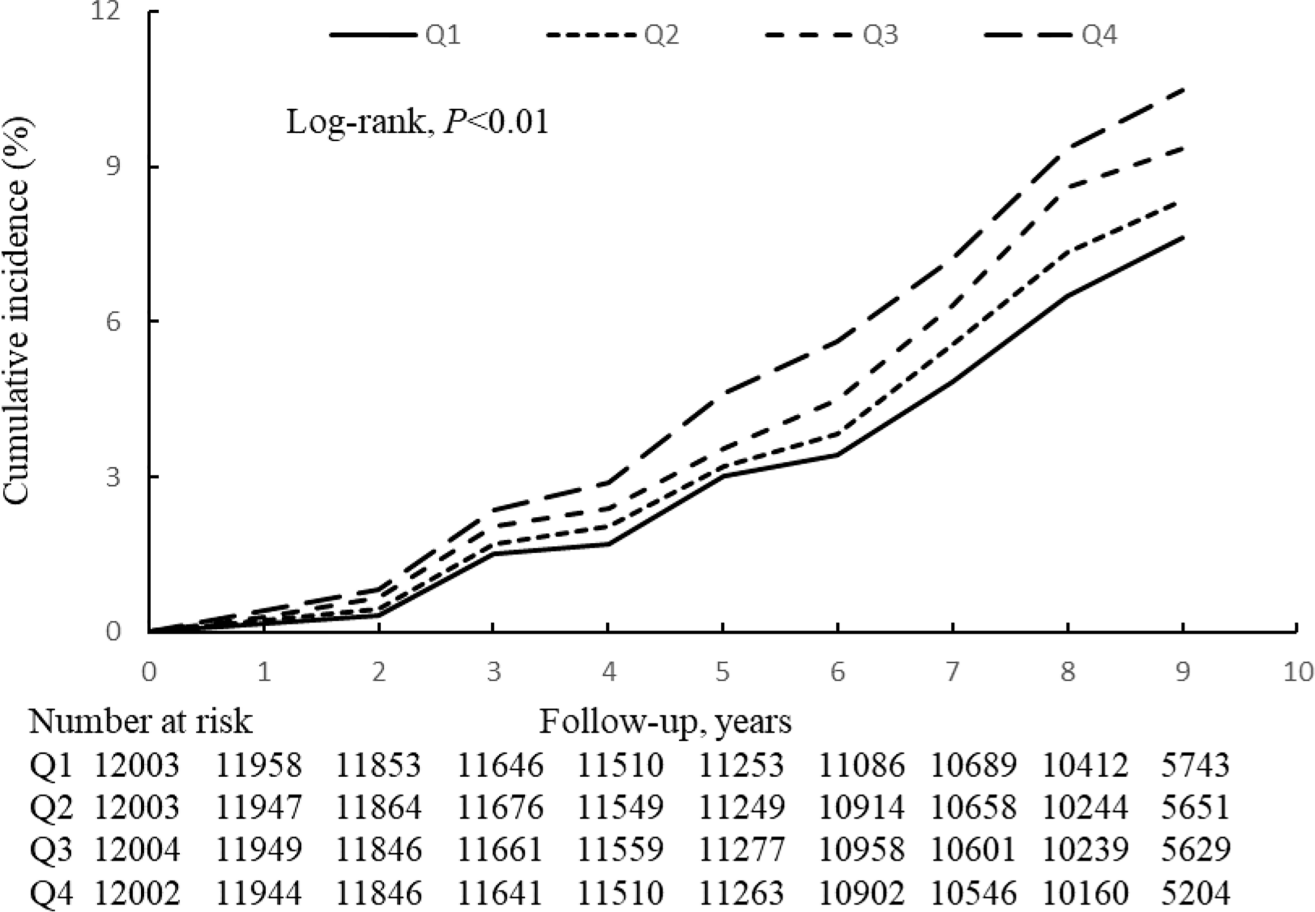
Kaplan-Meier incidence rate of diabetes by TyG-index variability (SD).

**Figure 3:**
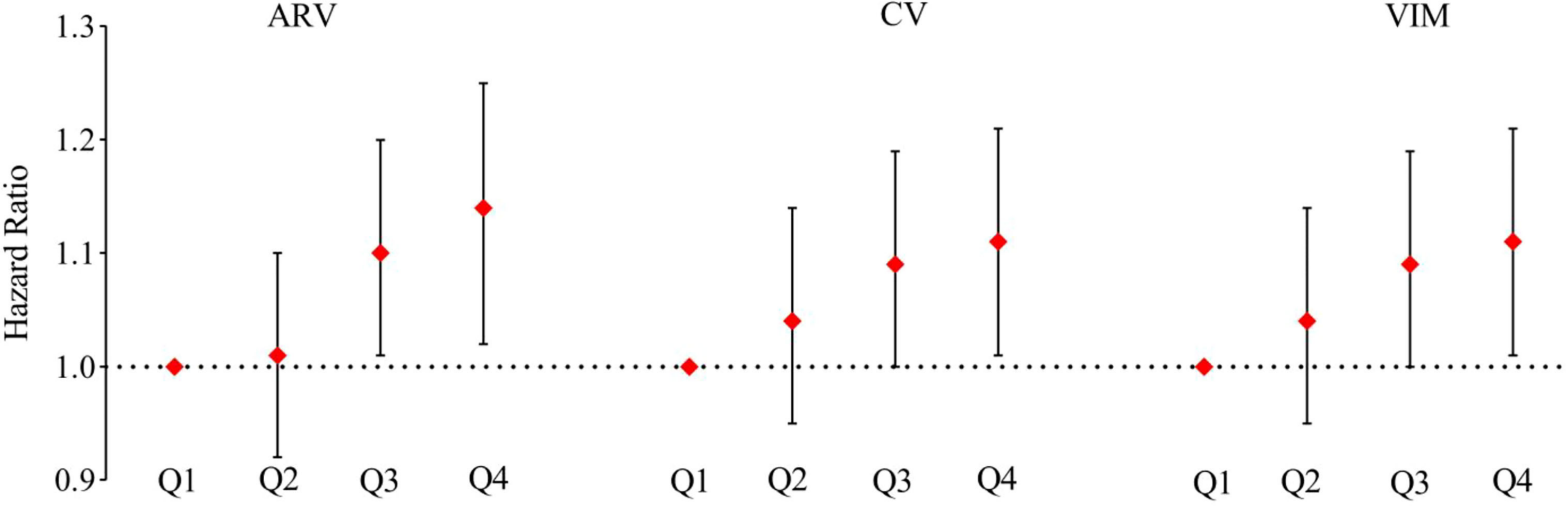
Sensitivity analysis of the association of TyG index variability with incident diabetes according to other indices of TyG-index variability (Average Real Variability, Coefficient of Variation, Variability Independent of the Mean) replacing Standard Deviation the in all the models. Model adjusted for age, sex, LDL-C, HDL-C, hs-CRP, BMI, smoking status, alcohol consumption status, physical exercise habits, educational level, hypertension, the use of lipid-lowering drugs, and TyG index.

During the mean follow-up period of 8.95 years (95% confidence interval (CI) 8.48–9.29 years), 4,055 (8.45%) of the participants developed diabetes. The incidence of diabetes increased with increasing TyG-index variability quartile, from 8.80 in Q1 to 11.70 per 1,000 person-years in Q4 (**Tables 2**, **3**). **Figure 2** shows that the participants in Q4 had a higher cumulative incidence of diabetes than those in Q1 (log-rank test, *P*<0.01). **Tables 2**, **3** shows the risk of incident diabetes according to the category of TyG-index variability, and the hazard ratio (HR) (95% CI) for Q4 *versus* Q1 was 1.34 (1.23–1.47) after adjustment for potential confounding factors. This association remained even after adjustment for the baseline TyG-index (HR 1.18, 95% CI 1.08–1.29). Each 1-SD increase in the SD of TyG-index variability was associated with a 4% higher risk of diabetes (HR 1.04, 95% CI, 1.01–1.07). In addition, similar results were obtained when the variability in the TyG-index was assessed using the ARV, CV, and VIM (**Figure 3**).

**Table 3:** Hazard ratios and 95% Confidence intervals of incident diabetes of TyG index variability (SD).

	Case/Total	Incidence rate, per 1000 person-years	Model 1	Model 2	Model 3
Q1	891/12003	8.80	1.00	1.00	1.00
Q2	933/12003	9.18	1.05(0.96,1.15)	1.04(0.95,1.14)	1.04(0.95,1.14)
Q3	1042/12004	10.28	1.19(1.09,1.30)	1.16(1.06,1.27)	1.13(1.03,1.24)
Q4	1189/12003	11.70	1.39(1.27,1.51)	1.34(1.23,1.47)	1.18(1.08,1.29)
1-SD increase (0.22)			1.20(1.09,1.15)	1.11(1.08,1.15)	1.04(1.01,1.07)
*P* for Trend			<0.0001	<0.0001	<0.0001

Model 1: adjusted for age (continuous variable, years) and sex (categorical variable, men or women) in 2010.

Model2: included variables in model 1 and further LDL-C (as a continuous variable), HDL-C (as a continuous variable), hs-CRP (as a continuous variable), BMI (as a continuous variable), smoking status (as a categorical variable, yes or no), alcohol consumption status (as a categorical variable, yes or no), physical exercise habits (as a categorical variable, yes or no), educational level (as a categorical variable, high school or above vs. below high school level), hypertension (as a categorical variable, yes or no), and the use of lipid-lowering drugs (as a categorical variable, yes or no) in 2010.

Model 3: included variables in model 2 and further the TyG index (continuous variable) in baseline.

### Results of the stratified and sensitivity analyses

**Table 4** shows the results of the stratified analyses. In general, high TyG-index variability (group Q4) was significantly associated with a higher risk of diabetes across the various stratified groups. There were no significant effects of age, sex, or the slope of the change in the TyG index on the association between TyG-index variation and incident diabetes.

**Table 4:** Hazard ratios and 95% Confidence intervals of incident of TyG index in subgroup ratio variability (SD).

	Age (*P* for interaction 0.33)		Sex (*P* for interaction 0.16)		TyG index change slop(*P* for interaction 0.74)	
	<45 years	≥ 45 years	Female	Male	>0	≤0
**Quartiles**						
Q1	1.00	1.00	1.00	1.00	1.00	1.00
Q2	1.09 (0.92,1.30)	1.02 (0.93,1.12)	1.07 (0.88,1.30)	1.03 (0.93,1.15)	0.93 (0.81,1.05)	1.16 (1.01,1.32)
Q3	1.23 (1.06,1.50)	1.07 (0.96,1.19)	1.18 (0.98,1.43)	1.11 (1.01,1.24)	0.98 (0.94,1.01)	1.28 (1.13,1.46)
Q4	1.25 (1.06,1.46)	1.11 (1.00,1.24)	1.36 (1.12,1.65)	1.14 (1.02,1.25)	1.06 (1.01,1.11)	1.50 (1.32,1.70)
*P* for trend	<0.01	0.04	0.02	<0.01	<0.01	<0.01

Adjusted for age (continuous variable, years), sex (categorical variable, men or women), LDL-C (as a continuous variable), HDL-C (as a continuous variable), hs-CRP (as a continuous variable), BMI (as a continuous variable), smoking status (as a categorical variable, yes or no), alcohol consumption status (as a categorical variable, yes or no), physical exercise habits (as a categorical variable, yes or no), educational level (as a categorical variable, high school or above vs. below high school level), hypertension (as a categorical variable, yes or no), the use of lipid-lowering drugs (as a categorical variable, yes or no) in 2010, and TyG index (continuous variable) in baseline.

With respect to the sensitivity analyses, the results of excluding outcome events occurring within the first year of follow-up, individuals taking lipid-lowering or antihypertensive medication, or individuals with TG ≥ 2.3 mmol/L at baseline were consistent with the results of the principal analysis. Because the SD may depend upon the mean value for each person, we also reanalyzed the data using other indices of TyG-index variability (ARV, CV, and VIM) in place of SD, but the findings were unaffected (**Table 5**).

**Table 5:** Sensitivity analysis of the association of TyG index variability with incident diabetes.

	Sensitivity analysis 1	Sensitivity analysis 2	Sensitivity analysis 3	Sensitivity analysis 4
Q1	1.00	1.00	1.00	1.00
Q2	1.04 (0.95,1.14)	1.02 (0.93,1.12)	1.11 (0.98,1.26)	1.03 (0.94,1.13)
Q3	1.13 (1.03,1.24)	1.13 (1.03,1.24)	1.17 (1.03,1.33)	1.13 (1.03,1.23)
Q4	1.18 (1.08,1.29)	1.19 (1.06,1.28)	1.42 (1.25,1.62)	1.22 (1.12,1.34)
*P* for Trend	<0.0001	0.0002	<0.0001	<0.0001

Adjusted for age (continuous variable, years), sex (categorical variable, men or women), LDL-C (as a continuous variable), HDL-C (as a continuous variable), hs-CRP (as a continuous variable), BMI (as a continuous variable), smoking status (as a categorical variable, yes or no), alcohol consumption status (as a categorical variable, yes or no), physical exercise habits (as a categorical variable, yes or no), educational level (as a categorical variable, high school or above vs. below high school level), hypertension (as a categorical variable, yes or no), the use of lipid-lowering drugs (as a categorical variable, yes or no, except sensitivity analysis 2), and TyG index (continuous variable) in baseline.

Sensitivity analysis 1: the exclusion of participants in whom diabetes developed within the first year of follow-up.

Sensitivity analysis 2: the exclusion of participants who were taking lipid-lowering or antihypertensive medication.

Sensitivity analysis 3: the exclusion of participants with TG ≥ 2.3 mmol/L in baseline.

Sensitivity analysis 4: adjusting for the baseline TG and FBG and without the inclusion of the baseline TyG-index.

## Discussion

In the present study, we have shown that high variability in the TyG-index is an independent risk factor for incident diabetes, even in individuals who are not taking antihypertensive or lipid-lowering medication and do not have a TG concentration ≥ 2.3 mmol/L by means of a longitudinal cohort study.Several previous studies have evaluated the relationship of a single TyG-index value with diabetes in the general population (32–36). For example, a 9-year follow-up study showed that individuals with the highest TyG indexes were at a 2.30-fold higher risk of developing diabetes (37). In the China Health and Retirement Longitudinal Study, which involved 3.4 years of follow-up, every 1-SD increase in TyG index was associated with a 22% increase in the risk of developing diabetes (HR 1.22, 95% CI 1.14–1.31) in Chinese people of 45 years or above (36). In addition, TyG-index is positively associated with CVD in patients with diabetes (38). The results of the present study extend these findings by showing that visit-to-visit fluctuation in TyG-index is positively associated with the incidence of diabetes in the general population, independent of conventional risk factors for diabetes and the baseline TyG index. This implies that both the absolute value and the fluctuation in the TyG-index influence the risk of incident diabetes in the general population.

We have previously shown that the risk of diabetes is lower after antihypertensive and lipid-lowering therapy (39, 40). Therefore, we repeated the analysis after excluding individuals who were taking antihypertensive or lipid-lowering drugs, but this did not affect the findings. In addition, because metabolic abnormalities, including a high circulating TG concentration, increase the risk of diabetes (41), we excluded participants with TG ≥ 2.3 mmol/L, but the results obtained were similar. Therefore, our findings emphasize the importance of regular monitoring and the maintenance of an appropriate TyG-index to prevent diabetes in the general population, even in individuals who are not taking antihypertensive or lipid-lowering medication and in those who do not have a TG concentration ≥ 2.3 mmol/L.

Although the mechanism linking high TyG-index variability with the development of diabetes has not been identified, there are several possible candidates. First, TyG is an index created using the fasting TG concentration and FBG (17, 23); therefore, high variability in TyG may be derived from large fluctuations in serum TG and/or FBG, which are associated with vascular endothelial cell dysfunction, oxidative stress, and inflammation (42–45), all of which are key pathophysiological features of diabetes (46). In addition, β-cell dysfunction is a key defect in the pathogenesis of diabetes (47), and aberrant glucose and lipid metabolism can lead to the apoptosis of β cells (48), which causes a deterioration of glycemic control and ultimately the development of diabetes.

The strengths of the present study include that it represents the first assessment of the relationship between the fluctuation in TyG index between clinic visits and the risk of developing diabetes, performed using data from a large, prospective cohort study. However, the study also had some limitations. First, we did not distinguish type 1 and type 2 diabetes mellitus in the present study. However, the Chinese diabetes guidelines state that type 2 diabetes currently accounts for 95% of all cases of diabetes (29) and that type 2 diabetes is more common in older people. Given that the mean age of the study participants was 48.78 years, the present findings are likely to be largely representative of the risk type 2 diabetes. Second, the observational design of the study prevents the confirmation of a causal relationship between the variability in TyG index and diabetes. However, when we excluded individuals who developed diabetes within a year, the results were similar. Third, we did not assess the changes in blood glucose using other methods, such as the measurement of glycated hemoglobin or continuous blood glucose monitoring. Fourth, despite adjusting for potential risk factors for cardiovascular disease, because the study was an observational cohort study, other sources of residual or unmeasured confounding may still have existed, such as differences in diet.

In conclusion, we have shown that TyG-index variability is an independent risk factor for new-onset diabetes, which implies that TyG-index should be maintained to prevent the development of diabetes.

## Data availability statement

The raw data supporting the conclusions of this article will be made available by the corresponding author, without undue reservation.

## Ethics statement

The studies involving human participants were reviewed and approved by the Kailuan General Hospital Ethics Committee (approval number: 2006-05). The patients/participants provided their written informed consent to participate in this study.

## Author contributions

XW, and YaC wrote the main manuscript text and conceived and designed the study. ZH analyzed the data. ZCa, and XY carried out literature search. ZCh and GC were responsible for developing the first draft of the manuscript. LL, KW, and HZ were responsible for developing the second draft of the manuscript. SW and YoC performed the manuscript review. All authors contributed to the article and approved the submitted version.

## Funding

This work was supported by the National Natural Science Foundation of China (No. 81870312).

## Acknowledgments

We thank all the survey teams of the Kailuan study group for their contribution and the study participants who contributed their information.

## Conflict of interest

The authors declare that the research was conducted in the absence of any commercial or financial relationships that could be construed as a potential conflict of interest.

## Publisher’s note

All claims expressed in this article are solely those of the authors and do not necessarily represent those of their affiliated organizations, or those of the publisher, the editors and the reviewers. Any product that may be evaluated in this article, or claim that may be made by its manufacturer, is not guaranteed or endorsed by the publisher.
